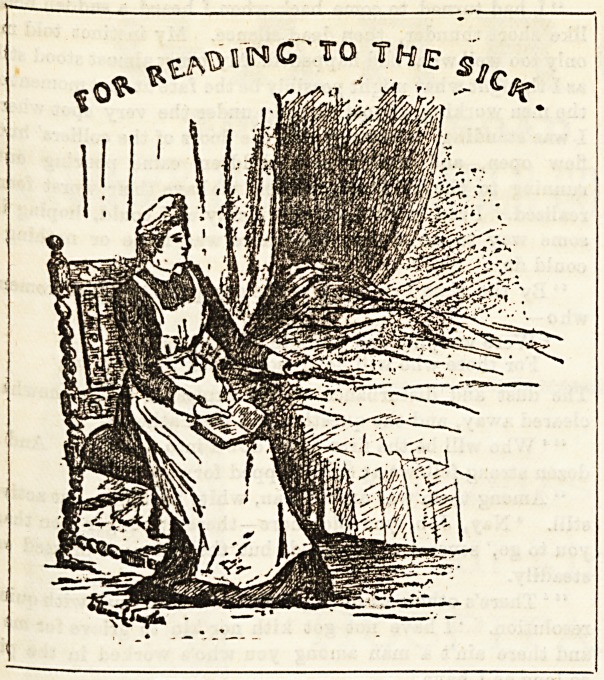# The Hospital Nursing Supplement

**Published:** 1892-07-23

**Authors:** 


### "The Hospital" Nursing Mirror.

Being the Extra Nursing Supplement of "The Hospital" Newspaper.

Contributions for this Supplement should be addressed to the Editor, The Hospital, 140, Strand, London, W.C., and should have the word "Nursing" plainly written in left-hand top corner of the envelope.

### En Passant.

REGISTRATION OF NURSES.—The hearing of the petitions for and against the application to grant a Royal Charter to the R.B.N.A. is likely to come before the Privy Council on or about Wednesday next, July 27th.

THE WARWICK NURSING ASSOCIATION.—This Association issues a second report, and shows that the work has taken root and is on its way to extension. Nurse Scott paid 3,488 visits during the year, and the year's expenses were £88 3s.; the work was exceptionally heavy owing to the influenza epidemic.

THE HOME OF COMFORT FOR EPILEPTICS.—An appeal appeared in the press last week on behalf of female epileptics, signed by the Earl of Meath, Sir Andrew Clark, Sir Dyce Duckworth, Dr. Thomas Hawksley, Dr. Napper, and Mr. Gibson. A house and grounds have been bought at New Godalming, and it is hoped that the public will now provide the means of maintenance for the inmates. Cheerful occupation is to be a marked feature in the establishment, and it is hoped that the work of the women will partly keep the home going. We have been talking of doing something for the sufferers from this malady so many years that one begins to doubt the consummation of any scheme to relieve them, but we hope soon to hear that the Committee of the Colony for Men and Boys have worked out their plan of action successfully also, and are ready to set to work.

NURSES CO-OPERATION. — The quarterly meeting of the General Committee took place on the 14th inst., and a very satisfactory report was received of the progress made during the last three months. Nurses have been sent to 458 cases since the last meeting, and the financial out-look is altogether most hopeful. Now that holidays are the order of the day, we are glad to think that the co-operation nurses will be able to indulge themselves in long and restful vacations during the next two months when their services are in less demand. Those who receive good pay, with only the deduction of a small percentage, are wise in devoting a portion of their earnings to securing the kind of holiday which will best fit them for returning in perfect health to those duties which can only be conscientiously fulfilled by nurses in sound condition.

"TRUTH" AND THE "LONDON."—We cull this extract from last week's number. Comment is needless: — "By the way, 'An Admirer of the Nursing Profession' has taken the trouble to write (anonymously) to inform me that I am 'very unfair' in having said nothing about that portion of the Report which deals with nursing. It was clearly stated at the outset of my remarks that I proposed only to deal with such points as had already been under discussion in *Truth*. What there was 'unfair' in this I am at a loss to understand. I am, of course, perfectly well aware, as I suppose everybody else is, that there is great room for improvement in the treatment of hospital nurses, and when it comes to giving effect to the recommendations of the Report, I shall take care that this portion of it is not overlooked. But it is also right to remember that the Report distinctly negatived the most serious allegations that have been made on this subject—those relating to the London Hospital."

THE LONDON HOSPITAL EXAMINATION.—The annual examination of the nurses gave the following results:—1st prize, Miss Edith Eastmond; 2nd prize, Miss Isabella Lister; 3rd prize, Miss Mary Issard. Five nurse gained honorary certificates, namely, Miss Gertrude Hicks Miss Anne Phelps, Miss Blanche Austin, Miss Margaret Harrison, and Miss Marion Daw. The examinations took place on June 30th, and July 1st and 2nd.

TEA AND TALK.—In spite of pouring rain, a very pleasant gathering met at the Trained Nurses' Club, in Buckingham Street, on Monday last. Mrs. Henry Smith, the President of the Midwives' Institute, was "at home," and a good many supporters of the Registration Bill were there discussing subjects various. We noticed Miss Paget, wearing the gold badge of the Q.V.J.I.N.; Sister Katherine, from Plaistow; Miss Atkinson, Miss Manley, Mr. and Mrs. G. Q. Roberts, Miss Brierly, and Mrs. Nichol busy dispensing tea and coffee; Miss Honnor Morten, and many others interested directly or indirectly with nurses and their work

SHORT ITEMS.—The Middlesborough nurses have paid 657 visits during the month of June.—The Aberdeen district nurse, Miss Armstrong, has attended 26 cases since May 20th, several of them requiring two and three visits a day. Funds are required to extend the work.—Nurse Eversfield, the district nurse at Bradninch, Devon, has resigned her post.—We are requested to state that the name of the Ballymena district nurse is Knight, and not O'Niel.—We have had a great many Sunday admissions given us for the Zoo and the Botanical Gardens all of which have been used by hospital nurses; we will gladly distribute any more.—Will our readers remember our Christmas parcels, and get their friends to help when possible?

UNIFORM.—We have seen and heard it asserted lately that the truly feminine joy women take in adorning themselves is the reason which is fast causing the real meaning of many nurses' uniforms to become lost amongst us, that nurses like other women like variety, and so the simple severity we were accustomed to in our nurses' dress is giving way to variations which tend to lessen its dignity. There are very few remarks which fail to point part of a moral at least, and some of the so-called uniforms are terrors to behold. Any good nurse realizes fully the value of a plain workmanlike garment, which is becoming and useful at the same time, and she avoids trimming and a long gown from a practical point of view. And, besides, it is the fitness of things that appeals to her; the tending of sick folk and the wearing of furbelows do not seem to go well together.

THREE YEARS' TRAINING.—"Some Thoughts about Nursing," by Oswald Browne, are kindly sent us by the author, and they are all of them good thoughts. How often women write to us asking how soon they can gain a certificate and if they must really spend three years in training. Here is a "thought" on the subject: "Constant familiar intercourse with the sick day after day, night after night; this is your immense advantage now. It is the great meaning of these three years that you spend in hospital work. The opportunity of gaining a sound practical experience is unique, and yet how short it is; and once passed it will never again recur to you. How all-important it is that you make the right use of and turn to the very best account the opportunity that is given. Here where the sick are gathered all about us every day is our best school of medicine and of nursing."

### Ventilation, Disinfection, and Diet.

By P. Caldwell Smith, M.D.

XV.—DIET AND DIETARIES—*(continued)*.

Milk—Its Composition—Necessity for Boiling before Use—Preserved Milk—Butter Milk—Butter—Margarine—Cheese—Bread—Oatmeal.

The next article of diet is one which could not possibly be done without. I refer to milk. It is the natural food of all human beings, and contains all the substances necessary for the growth and maintenance of the system. The composition of milk varies largely according to the animal from which it is derived, and even in the same animal the composition varies at different periods.

Take for example cows' milk, which is the most common form used. In 100 parts we find 86·87 of water, nitrogenous matter from 4 to 4·6, sugar 4·2 to 5·2, fat 3·5 to 3·9, and ash ·7 to ·8. Comparing it with other milks water is least in cows' milk, and the amount of nitrogenous material, that is the casein or curd and albumen, is greatest in cows' milk, as also amount of ash, while milk sugar is greatest in human milk. The table shows why cows' milk has to be diluted and sweetened when given to infants, as it contains more of all the solid constituents except the milk sugar. To imitate human milk then, cows' milk requires to be diluted with $\frac{1}{3}$ of water, and sweetened by means of $\frac{1}{4}$ oz. sugar to the pint of milk. It is always best to use sugar of milk, which may be purchased at any chemist's, rather than ordinary sugar. Milk, however suitable for children, is not so suitable for adults as a diet, as the amount taken would require to be great, and besides, there is a large amount of waste, as much as six to ten per cent. passes away without being assimilated. This is contrary to popular opinion regarding milk, but it has been proved by repeated experiments.

Milk before it is used should always be boiled. This should be a rule, without exception, applying both to children and adults in health and disease. The great objection to boiled milk is that it loses its pleasant taste, but this can to a large extent be avoided, if the milk be boiled at once when fresh from the cow, or as soon as it is received. The reasons for this are that boiling destroys the germs of disease which may exist in the milk, such as the germs of consumption, scarlet fever, typhoid fever, and diphtheria. A large number of epidemics of the three last diseases have been traced to infected milk, and if the milk had been boiled before being drunk these would never have arisen.

Preserved milk is now very much used. There are two varieties of it, the sweetened and the unsweetened, the latter being the best. It is useful when fresh cows' milk cannot be obtained, but it is questionable if it should, at least in the sweetened form, be used so largely as a diet for infants. The sweetened is easily taken by infants because of the amount of sugar it contains, and although children fed on this look fat, yet there is a want of firmness about them and they lack staying power, or more exactly they have not the same power of resisting disease.

Butter milk is an article of diet which is not sufficiently appreciated by the public. It is generally regarded as not containing much nutrition, but this is an error, as in the making of butter only the fat is extracted from the sweet milk, while the nitrogenous proportion and the sugar remain pretty much the same. It also frequently happens that small particles of butter are left in the milk, and when this is the case, it is often as valuable a food as sweet milk. It is of use when there is a deficiency of other kinds of nitrogenous food, as *e.g.* beef and mutton.

Butter as you know is obtained from milk, in which it exists as small globules. A very small amount of some of the other constituents of milk get into the butter as casein or curd, some of the mineral matter and water. When butter is melted in a glass heater or tube the casein falls to the bottom, the amount of this present in butter being about 2¼ per cent., while there is generally about 12 per cent. water. This is the favourite way of administering fat for two reasons, because it is the most digestible form, and because the flavour is more pleasant than most other forms of fat. Margarine is now very commonly used instead of butter, owing to its lower price, and the only fault that can be found with it dietetically is that it is slightly less digestible than pure butter. Good margarine is a long way better than bad butter, and as most manufacturers now send into the markets a very good margarine, it is better for those whose means are moderate to use this than the cheaper qualities of butter.

Cheese, although not by any means to be recommended in invalid's diet, yet is one of the most important foods we have for the working classes, and for these reasons: It is cheap, and contains a large amount of nitrogenous material in small bulk. Of course, different cheeses vary much in composition, according as they are made from skim milk or sweet milk. Made from skim milk, they contain a larger amount of nitrogenous matter, but, as would be expected, a smaller amount of fat.

Bread.—We now come to the consideration of the most important of all our foods. This may be of various kinds, as wheaten bread, rye bread, &c., but we in this country always mean bread made from wheaten flour. Bread, then, is wheaten flour made into a paste with water, $CO_2$ formed in it in different ways, either by the action of yeast, or by $CO_2$ being forced into the dough, and then baked.

The reasons why wheaten bread is used so commonly are obvious. It is produced easily and cheaply, contains all the elements necessary for the human body, is easily ground and baked, and has a taste and flavour which does not readily lead to satiety or disgust. It is used very much more in temperate climates, and to this may be due the bodily and mental vigour of the inhabitants of these regions.

Yeast is used to cause fermentation in flour and evolution of $CO_2$. This $CO_2$ permeates through the bread, and is the cause of its rising. Lately, by a patent system, this $Co_2$ is forced through the dough, and most machine-made bread is now made thus. All the baking-powders used are simply for the purpose of evolving this $Co_2$, on water being added.

Bread is more digestible than meat when made from fine wheaten flour, but rye bread and bran bread are much less digestible. The amount of residue which escapes is almost double in bran bread to what it is in ordinary wheaten bread, while it is apt to irritate the internal coating of the bowels and cause diarrhœa. Oatmeal is a very important food, and as porridge or brose has been used in Scotland as a main article of diet for some centuries, although in this last decade of the nineteenth century its use has died out to a very large extent among the working classes. It contains all the elements of nutrition, a very large amount of nitrogenous matters, and, for a cereal, a large amount of mineral matter. It has also a larger proportion of fat than ordinary flour, and one could exist on oatmeal alone better than on flour alone.

### Appointment.

The General Hospital Birmingham.—Miss C. A. Allen, who has been Matron at the Cumberland Infirmary, Carlisle, for four years and a-half, has been elected Matron at the General Hospital, Birmingham. Miss Allen trained at King's College Hospital, and has held the post of sister at King's College, at the Women's Hospital, Soho, and at Netley, and she was also night superintendent at the Children's Hospital, Pendlebury.

### Staff Nurses.

THEY may be called either "charge," "staff," or "head" nurses, but, whatever the name, the position itself is always an important one. The degree of responsibility attached to the post varies with the hospital, for in some there is a "sister" over each ward, to whom, of course, the nurse is subservient; in other places there is but one "sister" over several wards, and there the head-nurse in each has naturally more individual responsibility, whilst in some hospitals where there exists no such rank as "sister," the staff-nurse, although, of course, under the matron's authority, has sole charge of her ward, and the management of it includes a great deal, besides the care of the patients. They have the first consideration, both as regards the strict carrying out of all the doctor's orders for them; and again in the numerous details of daily and hourly care, which are summed up in the words, "skilled nursing." But besides the personal supervision of her patients, the charge nurse has to superintend the ward itself, from the diets, which it is her daily duty to distribute (and when they are in any way unsatisfactory, it is equally her duty to report that fact in the proper quarter), down to the regular scrubbing of the floors by the women engaged for this special work, who often, by the way, need a great deal of looking after. These are only specimens of the work which lies in her hands. When there is a "Sister," she, of course, shares and over-sees many of these details in addition to her other duties, but even then, a good nurse, in permanent charge of a ward, finds her own work more likely to increase than to decrease as time goes on. The name "staff" nurse sounds so essentially descriptive of the place she should hold: as the "sister's" "stay" she should also prove her best helper, and she becomes invaluable to the doctors, who see the changing probationers with indifference so long as the nurse, on whose intelligent service they are accustomed to depend, does not fail them.

But if any of these probationers, especially one of those birds of passage who drift into hospitals for only a short sojourn, and have therefore no true judgment of the relative value of things, were asked to describe the staff nurse, she would probably give a graphic summary, as, for instance, "The nurse of my ward is quite horrid! She is so dreadfully strict, everything must be done just in her own precise way! She is very good to the patients, certainly, but she is hard on the pros.!" Or, another critical young nurse will say, "Well, my 'staff' is very fair, she divides the work honestly and does not mind teaching, but she is so particular; she made me wash a feeding-cup twice over yesterday"; or, "She put a poultice I had made behind the fire, saying that it was not fit to be used for one of *her* patients, such nonsense!" or again, a more just girl may be heard to say, "Perhaps, after all, we pros. *do* try the staffs' nearly as much as they worry us."

Yes, of the last fact there is but little doubt, and it certainly is much to a trained nurse's credit when she greets each new-comer pleasantly, and starts her courteously in the way she should go. A kind word received on first arrival at hospital will dwell for years in a probationer's memory, and will often make of her a grateful and faithful worker. One valuable ward nurse, with whom many recollections of our own early pro. days are associated, was admired immensely by the juniors for her dainty professional appearance, as well as for her excellent general management, but her sharp tongue gained her many enemies. For instance, an anxious new-comer nervously demanding "What shall I do next," at a particularly busy moment, received the crushing rejoinder, "Well, I think you had better sit down and fold your hands, until you see something that wants doing." Such ill-manners are never excusable and, of course, always show a lamentable want of self-control. To "bear and forbear" is never more necessary than in the relations between staff and assistant.

The "Sister," when present, gives the tone to her ward, but this is maintained or destroyed, to a great extent, by the nurse.

Why do so many thoroughly satisfactory probationers prove failures when they blossom into nurses? Is the increase of responsibility too much for them to support with dignity? or is there some other reason for their want of success? Occasionally an injudicious matron or sister is to blame for making an undue fuss over the newly appointed nurse, and so helping forward an over-valuation of herself, before she has proved worthy of commendation. But, of course, when the matron is wise and the sister non-existent, this explanation fails. It is well that a charge nurse should realise that her own knowledge was only acquired through several years of experience, and also by the careful and patient training of those under whom she was placed, and her finish and polish were not gained in a day. It therefore behoves her to be equally long-suffering with the batch of raw material out of which the trained nurses of the future must be evolved.

### Hints on Hands.

WE cannot help wishing that nurses would attach more importance to the condition of their hands, for, unwearied as they are in the care they bestow upon their patient's skin, they certainly very often pay too little attention to their own. We sometimes see a nurse without gloves climbing up the staircase of an omnibus, and letting her fingers go into most unnecessary contact with a rail which is free to all comers. A few women manage to pass through their training without any serious damage to their hands, but such a feat is difficult of accomplishment, for there are many obstacles in the way. The dust and grime which attend the care of fires, even when the cleaning of the grates is the wardmaid's duty, and the constant need for washing, often hastily, with no moment to spare for thorough drying of the fingers, these are drawbacks as trying as the hardness of the water and the hardening properties of carbolic lotion. Still, no nurse need say that it is impossible to overcome these obstacles, although we allow that it is very difficult to do so, but it can be done. Let every probationer make up her mind to save her hands, not by doing less work with them, but by using more common sense and greater care in small ways.

Many of the "bad fingers" which we see or hear of could be altogether avoided by seasonable precautions. The hands should be made absolutely clean, by rubbing in plenty of water with good soap, then, when the skin has been partially dried, glycerine or lanoline should be lavishly used and firmly rubbed in, and the hands afterwards made perfectly dry. A few moments only are needed for the whole performance, which is as much a duty to the patient as to the nurse herself, for the former will certainly be benefited by the softer and more sensitive touch thus cultivated.

Glycerine does not suit all skins on account of the way in which it abstracts moisture, thereby tending to increase the dryness which is sometimes a characteristic, whilst lanoline is usually found to be a satisfactory application. Surely, on reflection, it is plain to all nurses that there can be no merit in "hard handedness," and the necessity that exists for their fingers being used in every variety of way, whether pleasant or unpleasant, does not absolve them from an obligation to adopt all reasonable precautions for escaping ill results.

### A Plan of Instruction in Invalid Cooking for Training Schools.

By Isabel A. Hampton, Superintendent of Nurses, and Principal of the Training School of the Johns Hopkins Hospital.

*(Concluded from page cix.)*

The organization of the cooking school resembles that of a ward with its head nurse and pupils, the teacher corresponding to the head nurse. Two pupils are sent to her for a month at a time. Their hours for duty are from 7.30 a.m. to 5.30 p m. The first hour and a-half of the day is spent in the private wards, where each pupil takes charge of the preparations for breakfast, makes the toast, arranges the trays daintily and gets everything in readiness for breakfast, which arrives from the general kitchen at eight o'clock. They then serve the trays and leave the wards at 9 a.m. going directly to the cooking school kitchen, which is a small room conveniently situated and easily accessible to all the wards. There they meet the teacher, and the day's instruction begins.

The course includes the general subject of the preparation of beef essences, beef teas, broiled meats, steaks, chops, and birds, gruels, porridges, mushes, drinks, jellies, toasts, soups, broths, oysters, eggs, potatoes, custards, sherbets, creams, frozen fruits, cordials, salads, koumiss, and all simple dishes, such as baked apples, plain boiled rice, also Graham gluten and white bread. The beef tea, chicken broth, and mutton broth for the use of the entire hospital are made each morning, and in addition practical demonstrations of the process of making dishes selected from the above schedule are given by the teacher every forenoon. The method of preparing about 150 different articles of sick diet is taught during the month, and each article is made at least three times by the pupils themselves. The greater part of the day's cooking is distributed among the various wards at noon.

Part of the afternoon hours are chiefly devoted to theoretical teaching, which includes, for example, talks on the effects of heat on food, the effect of cold, fire, the chemistry of foods, oxygen, the composition of air, water, the cooking of water, the mineral and organic matter in the same, albumen, and methods of serving food. All notes, lectures, and receipts are written out in full.

Towards the end of the month a practical test is given of the proficiency of each pupil, by requiring her to make as large a number of dishes as possible, without aid from either teacher or notes. An oral examination is given at the end of the course, and this is followed at the end of another month by a written test.

Diet Kitchen of the Johns Hopkins Hospital Training School for Nurses, Baltimore.

List of Dishes Prepared and Sent into the Wards for One Day, May 6th, 1892.

Ward D.
Beef broth, 1 pint.

Ward E.
Beef broth, 1 pint.
Snow pudding, 2 portions.
Beef juice, 6 oz.
Stewed prunes, 1 quart.
Mulled wine, 1 portion.

Ward F.
Beef broth, 2 pints.
Beef juice, 6 oz.
Restorative jelly, ½ pint.
Gluten bread, 1 loaf.

Ward G.
Beef broth, 1 pint.
Chicken broth, 1 pint.
Soft custard, 10 portions.
Sponge cake, 8 portions.

Ward H.
Chicken stew, 2 portions.
Beef broth, 1 pint.
Chicken broth, 1 pint.

Ward I.
Coffee jelly, 1 portion.
Velvet cream, 1 portion.
Potage a la Reine, 2 portions.

Ward C.
Beef broth, 2 pints.
Chicken broth, 2 pints.
Velvet cream, 3 portions.
Snow pudding, 3 portions.
Beef juice, 2 oz.
Oatmeal porridge, 10 portions.
Potage a la Reine, 3 portions.

Ward B.
Beef broth, 4 pints.
Chicken broth, 2 pints.
Oatmeal porridge, 12 portions.
Mock bisque soup, 6 portions.
Wine whey, 2 portions.
Mulled wine, 1 portion.
Broiled steak, 1 portions.

Selections from Written Examination Questions.

1.—Two potatoes are prepared for the table. One is cooked in water, the other baked in a temperature of 400 degrees Fahr. Which has the finest flavour? To what is this owing?

2.—Describe in detail the broiling of a chop. What other meats are cooked on the same principle?

3.—(*a*) What is the average percentage of starch in potatoes? (*b*) What is the percentage of fat in milk? (*c*) What is the composition of water?

4.—Give an account of the cooking and serving of a simple and nutritious breakfast for a person recovering from an illness.

5.—What is the most palatable and easily digested form of fat for an invalid?

6.—Which gives the least waste in digestion, animal or vegetable food?

7.—Trace the digestion of starch food, and show what connection there is between this process and previous preparation by cooking.

8.—(*a*) What should a healthful diet contain? (*b*) In what ways does food supply the wants of the body?

9.—A convalescent is left to your entire care for a week. You have unlimited supplies in the way of provisions. Write breakfast, dinner, and supper lists for each of the first three days of the week.

### Notes from Australia.

(*By Our Own Correspondent.*)

MELBOURNE, JUNE 7TH, 1892.

WHEN the influenza epidemic was raging here we thought the depression and gloom it brought amongst us was more than we could bear, but the commercial depression from which the community at large, including doctors and nurses, is suffering seems a good deal worse to endure.

Affairs are quieter at the Austin Hospital.

The Women's Hospital is much crowded, and some re-arrangement of the internal working of this hospital is under consideration.—The adjourned consideration of the request of the pupil nurses that the word "midwife" be substituted for "monthly nurses," as at present inserted in the certificates given to them by the Committee, has been resumed. After considerable discussion it has been decided to agree with the request that had been made, but in order that only thoroughly competent nurses should be provided with these certificates, a sub-committee is appointed, in conjunction with the midwifery staff, to consider the conditions under which midwives' certificates should be granted.

At the monthly meeting, in the last week in May, the complaint of overcrowding was brought forward by the Resident Medical Officer, who wrote stating that the midwifery department had been very crowded during the past week, so much so, that other wards had to do duty for after-treatment patients, and in some cases it had been necessary to crowd three patients into each of the small wards under the verandah. The beds and wards were being overworked, and if some means were not adopted of either increasing the accommodation or lessening the inrush of patients, puerperal septicæmia would sooner or later break out. Letters were also read from Drs. Adams and Fetherston in confirmation of the opinion expressed, and after some discussion it was decided to notify to intending patients, through the press, the advisability of communicating with the hospital authorities in order to learn whether there was a vacancy before presenting themselves for admission. To enable students of the Melbourne University to secure increased opportunities of clinical instruction, arrangements were made to enable a certain number of them to attend confinements at the houses of out-patients.

At the Homœopathic Hospital there is a new Matron, Miss Campbell. She had been in the hospital some years as Lady Superintendent; she now fills that post and the Matron's as well. The former Matron, Mrs. Muffit, is married to the former house-surgeon, Dr. Bonton.

Williams' trial and execution has, of course, renewed the question of crime being akin to insanity. I can remember knowing, many years ago, an eccentric child who had a fearful temper, and she was always excused because "she really must be insane." But where are we to draw the line in the matter of temper? Temper can be controlled, so can hysteria, if men and women will make themselves control it. For my part, I devoutly hope the day is far off when doctors will excuse temper and crime on the ground of insanity. The *Argus* had a capital article on "The Defence of Insanity, When is it Valid?" criticising the medical theory of insanity that if a prisoner's mind is diseased at all, he cannot be held to be responsible for criminal acts as though he were completely sane; that, in short, a partially diseased brain ought not to be regarded as capable of obeying ordinary moral laws. "This view," says the *Argus*, "attracts sympathy, but directly the question is faced practically, the difficulty of yielding to the medical theory becomes self-evident, where is that limit to be placed? Obviously there must, in the interests of the public safety, and for the sake of deterring crime, be some clear standard of the kind of insanity which can be successfully pleaded in the courts."

The ceremony of turning the first sod in connection with the Melbourne sewerage scheme was performed by His Excellency the Governor at Werribee, in the presence of some 400 invited guests. The guests were afterwards entertained by the Chairman of the Metropolitan Board of Works (Mr. Fitzgibbon) at a luncheon. The works will take several years to complete, and will involve a very large expenditure.

Snake-bite continues to be discussed in our papers without a complete solution of the best cure being come to. Complete cures and dead failures with both strychnine and ammonia are continually being reported, and the advocates of each cure seem equally sceptical of the other's good results, but it is much too serious a question to be left undecided, and a solution is earnestly desired.

### Presentation.

BRISTOL ROYAL INFIRMARY.—On the 7th inst. a pleasant and interesting gathering took place at the Bristol Royal Infirmary, when the Medical and Surgical Officers, Students, and Nursing Staff assembled to present the House Surgeon, Dr. James Swain, with tokens of their esteem and regard on his retirement from the position of Senior Resident Officer, and his advancement to the Honorary Surgical Staff of the Infirmary. The presentations consisted of a large inlaid writing table with drawers, which Dr. Benson offered from the past and present students, and a circular upholstered chair, with stationery cabinet and reading lamp, from the Nursing Staff. The senior Charge Nurse, Miss Langford, read an address in which, in the name of the Nursing Staff, she wished Dr. Swain every success and happiness in the future, and thanked him for his unfailing kindness and consideration to them on all occasions, and for having done so much to help them in their work. Small brass plates with the date of the presentation were fixed to the writing table and chair.

### Wants and Workers.

[Under this heading, we propose to try whether we can be useful to our readers in making the wants of some known to others who are willing to do what work they can to aid the great cause of curing and cheering the sick. Wants can only be inserted from those who are connected with some institution or association, or who are willing to have their full name and address printed.]

*The Lady Superintendent, Sanatorium, Bromsgrove*, would be greatly obliged for a carrying chair, of which the institution stands in need. Also for crochet or old woollen shawls of any kind for patients breakfasting in bed.

### Notes and Queries.

### Queries.

*Nurse E. B.*—Will any reader of this paper tell me if she knows of lodgings in Harwich, a bed-room and two sitting-rooms?

*M. W. C.*—Will somebody please tell me the publishers of the "Nursing of the Young," by James Marsh.

### Answers.

*Enquirer.*—The rules for the requirements for a district nurse of the Q.V.J.T.N. are: She shall have at least one year of training in a good hospital; she must have six months' training in an accredited institution for district nursing; and if called to work in the country she shall have at least three months' approved training in midwifery. You are quite wrong in supposing this institute employs untrained women; they aim at supplying well-trained nurses in every sense.

*Nurse M. M., Liverpool.*—The nursing scheme for India is not in any definite shape yet. If you watch our advertisements you may get something, but do not go abroad on chance of work. Unless you hear of work abroad privately, the only way to get it is through advertisement.

*Nurse M. E. S.*—We are quite willing to answer any question we can to help nurses on practical points, but such a column as you suggest would, we fear, hardly come within our scope; it would be rather liable to degenerate into quackery questions. Most of the points you mention could be found in a medical dictionary or a nursing w rk, but we will get some short articles on one or two of them; they are too important to be dismissed in a paragraph. A good dictionary would be of great help to you. Many thanks for suggestion, but you will easily see that difficulties might arise from it.

### Everybody's Opinion.

*[Correspondence on all subjects is invited, but we cannot in any way be responsible for the opinions expressed by our correspondents. No communications can be entertained if the name and address of the correspondent is not given, or unless one side of the paper only be written on.]*

### HOLIDAYS IN SWITZERLAND.

"Nurse E. M." writes: Some of your readers may be interested to hear of a charming holiday in Switzerland from which a fellow nurse and myself have just returned. We stayed two nights in Geneva, then, taking a small knapsack, we went by steam tramcar to Veyrier, and climbed up the Grand Salève to Monnetier, a tiny village nestling on the mountain, where the air is delicious, and the living moderate in price. We put up at Hôtel Trottet, the cleanliness and simplicity of which we much appreciated. We took all our meals out of doors, and no coffee ever tasted so good as that which we drank out of the quaint little bowls on the roughly-hewn tables. I cannot describe the joy of seeing Mont Blanc, from one's bedroom in the early freshness and sweetness of a summer morning. One day we went higher up the mountain to La Croix to obtain a better view of Mont Blanc, we seemed then for the first time to grasp some idea of its marble-like grandeur and majesty. Many evenings we spent walking about on the Petit Salève, gathering flowers and grasses, and trying to learn something of the herbs used by the old Paysannes in making tisanes. We had such a happy time at Monnetier, and we felt we had what we needed, a complete change for mind and body, and a sense of being out of, and a long way from the busy world, and there is nothing so refreshing as that to a tired nurse. Now we are ready and anxious to take up our work again—but such an impression has this real holiday made upon me, that I want other weary nurses to know of and become acquainted with my arcadia.

### THE MATRONSHIP OF LONGTON COTTAGE HOSPITAL.

Mr. Thomas Blair writes from Normacot, Stoke-on-Trent: A "Medical Correspondent" makes a perfectly correct statement of facts in the last issue of your paper, but I really cannot understand what he (or she) considers ought to be apologised for, or what further explanation anyone has a right to expect. Then, as to it being dishonourable, wrong, or unfair to re-elect the former holder of the post, perhaps you may be able to explain how and why this is so, and also how the rejected candidates are affected differently by the appointment of the former Matron than they would have been had anyone else been appointed. And now, Sir, may I ask why you consider this incident unique of its kind? Surely there must be some other reason besides that of a lady having changed her mind—which certainly does not appear to me strikingly unique; but, at any rate, I fail to see anything in the circumstances more so. I much regret feeling compelled to say that your assertion that a hoax has been practised upon 54 ladies is most distinctly untrue, and has not the slightest foundation in fact; neither has your further assertion that the ladies certainly have not received the reasonable consideration they deserved.

[Mr. Blair seems to be unaware of the custom of notifying in an advertisement for a vacant post that a junior member of the staff or the *locum tenens* will apply for it, when such is the case. This intimation enables everyone to understand that the appointment is not an open one in the ordinary sense of that term. The Longton incident is unique in the fact that where an officer has resigned, the resignation accepted, and the vacancy advertised, such proceedings as those at Longton have been heretofore held to be impossible amongst a committee of gentlemen.—Ed. T. H.]

### HOSPITAL AND ASYLUM NURSES.

Dr. Greene, Berrywood Asylum, writes: "I believe your interesting journal has a fair circulation in our county asylums, and it is rather curious to note that it seems to keep this circulation in spite of misrepresentations and slurs cast on the nursing staff; but there is a possibility of going too far. You lately told us that 'the dearest wish of your heart was to see the dying lunatic properly nursed.' Contrasts have more than once been drawn between hospital nurses and asylum nurses, and the former have had the preference. In your last issue you say that 'forty years ago hospital nursing was in as bad or worse a condition than asylum nursing now is.' And further on you reach the climax when you allow your reviewer to write that 'not a ray of enthusiastic light has directly penetrated their gloom.' In support of these statements made anonymously, and in transatlantic English, you quote individual opinions written probably twenty years ago. I think I know something of hospital nursing, and I am sure I know something of asylum nursing; and, taking them all round, I believe that the asylum nurse is superior in many ways to the hospital nurse. Considering the nature of the duties which have to be performed in asylums, the present asylum nurse is better than the present hospital nurse, and if tried in asylums the latter frequently break down. It may be that asylums are not yet perfect, and that improvements are required; but you may feel assured that no medical superintendent will move in the direction shown by the hospitals. Indeed, it could be proved that things are done in the nursing and in the management of hospitals that would not be tolerated for a day in our county asylums; and I am certain that if the hospitals copied the asylums it would be better alike for their subscribers and for their patients."

### "ONE WHO WANTS TO KNOW."

"A 'Trained' Inquirer" writes: What with nursing papers, pamphlets, and petitions, a quiet, old-fashioned nurse, trained in the early days of the schools which first countenanced "skilled nursing," hardly knows now what all these printed pages demand from her. Once upon a time we thought that women knew a great deal after a few months' "hospital work," but that idea did not last long, and we soon began to find that two or three years of regular training increased our value to the patients and the public. And lately most of us find that we cannot get any appointment worth taking unless we hold proofs of this full training, and those institutions which are established, not to merely make the manageress a good income, but to insure fair profits and protection to the nurses, are all making the hard and fast rule that complete training, as well as experience, are indispensable. I always read the notices of the grand things which the R.B.N.A. promises to nurses, but I cannot always see how they can benefit us; and there was an extraordinary decision published in connexion with the last meeting to the following effect: "That nurses who were early members . . . shall be allowed to be eligible for registration . . . ." Now, please explain, Mr. Editor, if you can spare the time, how a decision to register "early members," some of whom have been proved to have had hardly any training at all, can be reconciled with what is declared as a primary object of the Association, viz.: "The registration of nurses who have gone through not less than than three years' hospital training . . ." If we are to be helped by any of these much-talked-of associations, we should like to know what we are to believe, and also what we are to gain!

### THE MYSTERY OF PAIN.

LIFE is full of mystery; from the cradle to the tomb we are surrounded by things we cannot understand. "The wind bloweth where it listeth, and we hear the sound thereof, but cannot tell whence it cometh or whither it goeth," and it is the same with all the forces of nature, while of our own being who can answer the questions offhand, "Whence we came, and whither wending?"

Still anything mysterious has a great charm to most minds. To know there is something hidden from us excites our wonder and awe, while the lower feeling of curiosity bids us find out what there is behind the veil. No subject then can be more attractive to us than the mystery of pain, why it is in the world, why it affects us personally, and not our neighbour, and why it is measured out in such disproportion (so it seems to us), one man escaping slightly, while another is a martyr to agony. Of ourselves we can make no proximate guess on the subject; we simply wonder if it comes to us as a punishment, if we are more wicked than other people, or if God has sent a swift judgment on us for our sins. Sometimes we question whether we are the sport of chance which has given us somebody else's share as well as our own. In this last idea we are entirely wrong; our lives are ruled by an unerring God. Pain, however, in certain cases, there is no doubt, has been used for punishment, though all wise and great thinkers have come to the conclusion that suffering is corrective, that it is sent to purify our hearts and motives, and make our lives better and grander. In its full meaning the word "mystery" is something hidden from all but the initiated, that is those who have been taught the hidden secret. We may become initiated into the mystery of pain by looking on the Great Pattern of all sufferers, our blessed Lord himself. His life consisted of thirty years of poverty and labour in the sweat of His brow, followed by three more, into which the Jews crowded the contempt, hatred, and persecution of their ungrateful hearts, and it was brought to a close by a shameful death of agony. Who can fail to be "purged by the terror and pity," as the old Greeks have it, felt at the recital of such a fearful tragedy! But beyond this, as our Lord was made perfect through suffering, so our own pain, which is so cruel, this throbbing brow, these palsied limbs, are all leading us to the foot of the Cross, there to be washed and made white in the Blood of the Lamb. If we bear our trials with His patience, if we struggle and overcome in His strength, He will grant us to sit on His throne with Him, even as He also overcame and is set down with His Father in His Throne. Here is the true secret of pain.

"Beloved, think it not strange concerning the fiery trial which is to try you, as though some strange thing happened unto you. But rejoice, inasmuch as ye are partakers of Christ's sufferings; that when His glory shall be revealed ye may be glad also with exceeding joy.

### One Touch of Nature.

To a clergyman just come up to London for a few days, from a remote country parish far from the noise and bustle and distraction of a town life, what could be more unwelcome than to find that his cousin, the rector of a busy London parish, had met with an accident which kept him to his room, and was begging him to take his place that evening at a service in a ward of a neighbouring hospital. "I promised to take an evening service there every Wednesday, while the chaplain is away for his holiday. You won't mind old man, I know, will you? Quite a short service you know, something short and bright. Sick folk can't bear much preaching at; there are my books; I have looked out the hymns; sister will play the harmonium for you; I am so much obliged to you, George; I should have been sorry if the service had fallen through. The poor fellows don't have much variety to vary their long days and I generally manage to tell them something of what is going on in the outside world."

And the good weary man leaned back in his chair with a sigh of relief at having found someone to take his place. A quiet evening all to himself, surrounded by his dear book without fear of interruption, was a treat which did not often fall to his lot, although on this occasion it must be spent in one chair with his wounded leg carefully placed on a chair in front of him.

"But really, I hardly know what I shall say to them. Very glad to help you, of course, but I'm quite unprepared. I've never been inside a hospital in my life. What time did you say?"

"Oh! half-past five. You just have time to get there. Don't give them a large dose, George; just enough to make them want some more; that's the secret. Yes, I'm all right. I shall be glad to see you back, and hear how you found them."

Thus it happened that before Mr. Burton completely realised what he had undertaken, or had collected his ideas on the task before him, he found himself crossing the large bare hall of the hospital, and the porter's cheerful "This way, sir," sounding up the stairs ahead of him. The unfamiliar corridors seemed strangely long to him, and yet he could have wished them longer when he suddenly saw the words, "Dutton Ward," painted over a door, and knew he had reached his goal.

The bright kind Sister who advanced to meet him seemed, he imagined, a little disappointed to see a stranger, but she told him what servicetthey were accustomed to have in the ward, and hoped he would excuse her at the end of the hymn, as she had to take charge elsewhere.

The familiar strain of "Oh, Happy Band" was commenced—somewhat slowly, to suit weak lungs and short breath—but notwithstanding the very moderate pace, an old Daddy, who evidently was rather proud of his voice, but was too deaf to be guided by the wheezy tones of the much-used and long-suffering instrument, waxed slower and slower, and was finally left a full couple of bars behind—

"Where such a light affliction
Shall win so great a prize,"

sang the weak and faltering voices, and the last straggling notes were dying away before Mr. Burton had collected himself sufficiently to look round and take in the details of his surroundings. The ward seemed to him very long, but not so bare and dreary as he had pictured it to himself.

The daylight was closing in, and the flicker from the fire made a bright reflection in the highly-polished floor. The red counterpanes made cheerful patches of colour on either side of the ward, and the artistic arrangement made by the Sister of her few palm ferns and flowers on the central table helped to lend a pleasant look to the general effect.

Most of the patients had turned with curiosity to the door when the stranger chaplain entered; but one or two of the faces wore that look of patient endurance which told that the outward things of this life had been of little interest for men whose feeble energies were absorbed in the struggle with constant pain and weariness.

One face lying in a bed just in front of him struck Mr. Burton almost with a sense of awe. It was that of a very, very old man. His aged features, covered with countless wrinkles, wore an expression of absolute content and patient waiting. He seemed hardly conscious of his surroundings, but lay with a mysterious far-away look in his dim eyes, eyes which seemed already to see—

"Beyond the river which has no bridge,
Beyond to that land
Whose gardens and whose gallante walkes
Continuallie are greene;
Where growe such sweet and pleasant flowers
As nowhere else are seene."

In the next bed lay a young man—young in years only—young without the strength, the vitality, the hopefulness of youth; young, but with the enfeebled frame, the drawn and careworn look of old age. He looked one to whom life has given little, and now promised less. One defeated and worsted in the battle of life.

What should the messenger tell these men?

He had on leaving the house hurriedly slipt into his pocket a sermon, which had done duty more than once for his rustic congregation. He trusted it would not seem out of place here, or contain allusions which might appear incongruous.

Mr. Burton had to hold the print rather close to his eyes in the fading light, and read slowly and distinctly the first few pages, gathering courage as he went on. When the second head came, introduced with the words, "And now my brethren, do we not see here a singular manifestation?" he ventured to look up. Alas! not one face showed a trace of the faintest interest in or even comprehension of what he was reading to them.

"I generally manage to tell them something of what is going on outside," his friend had said.

"Give them enough to make them want some more." Excellent advice—but he had a very strong conviction that not only would no one want to hear more of his sermon, but that everyone (himself included) would feel relieved when it came to an end.

Mr. Burton laid the sermon down on the stand before him and paused. Most of the men turned their eyes on him to see the cause of his silence.

It lasted what seemed to them a long time, for Mr. Burton's thoughts had travelled far. Then he said:

"I have been thinking that we learn and remember little of a sermon like this compared to the lessons of daily life, which meet our eyes at every turn, whether we will see them or not. Let me tell you of what I saw a short time ago while I was on a visit to a coal mining district in the North. Some of you may know it. It is situated in the West of Yorkshire. The place is called Blayton Firs Colliery."

The young man turned round suddenly at the sound of the name, and kept his eyes fixed intently on the preacher.

"It was only a small colliery, and was thought to be nearly exhausted, but more than 200 men still worked there. One evening, as I wished to get a good view of the neighbourhood, I walked to a hill near Blayton Top, I think it was called."

"Aye, that's it," exclaimed the young man, now sitting up in bed, and leaning forward in order to catch every word which fell from the speaker's lips.

"The sun was setting, and its brilliant rays transformed the murky smoke which hung over the landscape into a glorious golden haze.

"I had turned to come back when I heard a sudden noise like short thunder, then dead silence. My instinct told me only too well what had happened. My heart almost stood still as I thought what might possibly be the fate at that moment of the men working almost directly under the very spot where I was standing. On every side the doors of the colliers' huts flew open, and women and children came pouring out, running to the pit's mouth then to have their worst fears realised. I ran to the spot as quickly as I could, hoping in some way to be of use, but there was little or nothing I could do.

"By the time I came up to the group of terrified women, who—

'Were weeping and wringing their hands
For those who will never come back to the town,'

The dust and disturbance of the explosion had somewhat cleared away, and the question had been asked—

"'Who will be the first to go down into the pit?' And a dozen strong fellows at once stepped forward.

"Among them was an old man, white with age, but active still. 'Nay, Job, you bide here—there's younger men than you to go,' several voices cried, but the old man pressed on steadily.

"'There's others as have got their own,' he said with quiet resolution. 'I have not got kith nor kin to grieve for me; and there ain't a man among you who's worked in the pit so long as I have.'

"And he stood quietly at the pit's mouth awaiting the signal to go, with his life in his hand, down into the darkness to give there, if need be, his own life in the attempt to save the lives imprisoned below."

Mr. Burton paused, his thoughts full of the harrowing sights which had met his eye, as he had waited hour after hour on that distressing scene, and had tried to speak a few words of comfort to those who seemed most to need it.

"I tell you this story," he continued, "because often and often since that time, the recollection of those brave determined men, so ready and so willing to give their lives for their comrades, counting, as St. Paul says, their lives as not dear unto themselves has done me more good than any written sermon could do," and then in a few plain words he summed up the lessons he wished them to learn from what he had said, and closed his address, followed to the last word by the close attention of the men.

"Eh, sir, that's father, sure enough."

Mr. Burton had to bend closely over the young man's bed to catch the words made almost inaudible through weakness and excitement. "Job Heyward, you said, sir; and he came up safe, did he? When did you say you were there, sir?"

And Mr. Burton had to describe the whole scene once more, and repeat, and repeat again, and to listen to the questions which the poor fellow seemed never tired of putting as to all that was said and done on that eventful evening. "Blayton Firs is where I was born, sir, and I have not been near it for twenty years. I ran away to sea when I was a bit of a boy, and many's the time I longed to have a sight of the old place."

"No kith nor kin to grieve for him did you say. Well! well! He's got one son, sure enough, and if ever I get better, sir, and out of this, I'll go straight back to old father if I've got to tramp the whole way;" and the weary head lay back on the pillows, too tired to say more than the last parting injunction—

"You'll write to him to-night, you said, sir, and thank you kindly. Tell him it won't be long before Jim's at home again, and he won't be in such a hurry to leave it again, he won't."

"Well, George, back again! I am very much obliged to you for all the trouble you have taken; I hope it has not tired you."

"Not at all," replied Mr. Burton, rather absently, as he hunted about for writing material. "I am very glad I went. It has given me real pleasure."

## Figures and Tables

**Figure f1:**
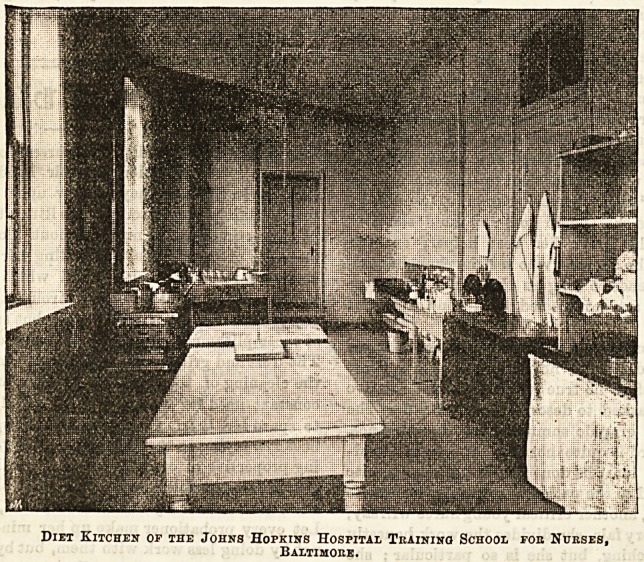


**Figure f2:**